# Is your mind set? – how are intra- and interpersonal competences dealt with in medical education? A multi-professional qualitative study

**DOI:** 10.1186/s12909-019-1748-y

**Published:** 2019-08-22

**Authors:** Lisa Lombardo, Jan Ehlers, Gabriele Lutz

**Affiliations:** 1 Institute for Didactics and Educational Research in Health Care, Department of Medicine, Faculty of Health, Witten/Herdecke University, Alfred-Herrhausen-Straße 50, 58455 Witten, Germany; 2 Integrated Curriculum for Anthroposophic Medicine (ICURAM), Medical Theory, Integrative and Anthroposophic Medicine, Department of Medicine, Faculty of Health, Witten/Herdecke University, Alfred-Herrhausen-Straße 50, 58455 Witten, Germany; 3 Chair for Didactics and Educational Research in Health Care, Department of Medicine, Faculty of Health, Witten/Herdecke University, Alfred-Herrhausen-Straße 50, 58455 Witten, Germany; 4 Integrated Curriculum for Anthroposophic Medicine (ICURAM), Chair for Medical Theory, Integrative and Anthroposophic Medicine, Department of Medicine, Faculty of Health, Witten / Herdecke University, Gerhard Kienle Weg 4, 58313 Herdecke, Germany; 5 Department of Psychosomatic Medicine, Gemeinschaftskrankenhaus Herdecke, Gerhard Kienle Weg 4, 58313 Herdecke, Germany

**Keywords:** Intra- and interpersonal competences, Medical education, Medical practice

## Abstract

**Background:**

Professional intrapersonal and interpersonal competences (IICs) form an important part of medical expertise but are given little attention during clinical training. In other professional fields such as psychotherapy, education and aviation, training in IICs is an integral part of education and practice. In medicine, IICs tend to actually decline during studies. To date it is unclear why IICs are given less attention in medicine, despite evidence for their importance in the treatment process.

In view of this, the study examined the role of IICs in the treatment process, the current situation of IIC training in medicine and, most importantly, the reasons for the comparatively low focus on IICs in the clinical training of medical students.

**Methods:**

Semi-structured interviews were carried out with 21 experts from a variety of medical specialties and non-medical professions that provide a training with a stronger focus on IIC development. The interviews were evaluated using *grounded theory*.

**Results:**

The experts confirmed the idea that IICs are an equally important component in the treatment process, along with medical knowledge and technical skills. They also described large differences between the IICs possessed by physicians but noted a general developmental need. The key shortcoming was perceived to be a deep-seated defensiveness towards learning from mistakes and deficits e.g. through reflection and feedback. The interaction of different factors that seem to be reasons for this defensiveness and perpetuate it were identified: lack of support in dealing with insecurities in the face of responsibility; the notion of medicine as a science with the categories of right and wrong answers; and a range of pressures arising from the setting, such as hierarchical, economic and competition pressures.

**Conclusion:**

Our study showed, that the defensive attitude towards learning from mistakes and deficits especially in the field of IICs appears to be a subtle but powerful obstacle for implementing IICs in medical training, in contrast to other professional fields. This obstacle is sustained by various underlying barrier factors. We therefore propose that changes should be made within a cultural transformation targeting this defensive mindset and culture and its presumed reasons.

**Electronic supplementary material:**

The online version of this article (10.1186/s12909-019-1748-y) contains supplementary material, which is available to authorized users.

## Background

Professional intrapersonal and interpersonal competences (IICs) form an important component of expertise in all medical specialties [1]. Intrapersonal competences are various skills and attitudes – such as self-reflection, self-care or self-regulation – which are important in dealing with yourself, while interpersonal competences are a requirement for the effective cooperation with others [1, 2]. Intrapersonal competences can be defined on the three levels of “intellectual openness, work ethic and conscientiousness, and positive core self-evaluation” that include “flexibility, initiative, appreciation for diversity, and metacognition” [1], whereas interpersonal competences comprise “communication, collaboration, responsibility and conflict resolution” in the domains of “team work and collaboration, and leadership” [1]. These IICs are often subsumed under the “artistic aspect” of medical practice, while medicine has repeatedly been described as a combination of “art” and “science” [3–5].

There are several curricular frameworks in medicine that already address IICs in their training objectives. For example, the ACGME cites “Interpersonal and Communication Skills” as well as “Professionalism” that enable physicians “to demonstrate a commitment to carrying out professional responsibilities, adherence to ethical principles, and sensitivity of diverse patient populations” as core competences [6]. Furthermore, the CanMEDs roles [2], which are used internationally in many medical schools as a template for designing the curriculum, were initially planned to integrate IICs explicitly in the role of the *person.* However, while developing the framework the *person* as an independent role has been lost, so that none of the remaining seven roles explicitly defines IICs, although they are identified as being necessary in every role [7]. In the Netherlands the role of the *reflector* was introduced into the CanMED’s model to foster IICs “in order to explicitly emphasise the importance of the ‘person’ of the trainee” [7]. In addition, the concept of *personal and professional development* and of professional identity formation cover approaches for increasing IICs [8].

Despite the existing theoretical concepts and some best practice examples for the implementation of communication skills trainings and reflective practice in personal professional development, the transfer of IICs to practice and therefore the implementation in daily clinical routine remains difficult in most places [9–12]. In practice IICs have to date often been taught “by chance, unchecked and individually” by medical role models who do not feel themselves competent to do this [13–17]. It is not only the further development of intrapersonal and interpersonal skills and attitudes which suffer from this situation: various studies show that there even seems to be a decline in these competences during the course of training, despite new training components such as communication skills training [18–20].

As a result of the recent shortcomings in teaching IICs, potentials might not be realised and negative consequences may arise in the areas of team cooperation, patient safety, training, work satisfaction, effectiveness and quality assurance [17, 21–24]. These shortcomings have already been recognised by some non-medical professions as well as by particular medical sectors. However, in other vocational fields, the implementation of structured trainings of IICs has often been done more comprehensively than in medicine. There are examples found in medical professions such as nursing, medical quality management and psychology, and in non-medical fields such as teaching and aviation as well as areas of management and IT [25–32].

In order to work on the difficulties of structured implementation, teaching and research often focus on the implementation of single competences. However, this does not seem to be sufficient for two reasons. On the one hand, the different IICs are seldom needed in an isolated fashion in practice. In fact, a professional attitude is formed by bringing different IICs into practice at the same time. On the other hand, the different IICs all lack a structured longitudinal implementation in medical curricula, continuing education and practice. This implementation, for example through structured feedback or opportunities for reflection within training and practice, seems to be counteracted by factors within the clinical environment. This observation strengthens the notion that there could be underlying “softer, less visible, aspects of health service organisations” [33] that hinder the implementation of IICs in general and which have not yet been identified. These aspects can have three layers: visible manifestations, shared ways of thinking and shared assumptions [33]. This raises the question of what the specific underlying aspects hindering the general implementation of IICs could be.

### The research questions

As capturing the status quo of a problem and identifying the factors which foster this problem are an important requirement for future approaches to change, our study should be viewed as an in-depth barrier analysis [34, 35]. While carrying out our research, our first goal was to gain insights into these softer, less visible barriers to the implementation of IICs in medical education, such as shared ways of thinking and deeper shared assumptions. The second goal, which will form the focus of a second publication, will be to build on this barrier analysis and work out detailed strategies and methods for change.

Given these theories, assumptions and goals, the research questions for this study were:

- Which role do intrapersonal and interpersonal competences play in the medical treatment process compared to knowledge and technical skills?
- What is the current situation of IIC training in medical curricula and continuing education?
- What reasons are perceived in medical training hindering the implementation of these competences?

## Methods

A qualitative research approach using semi-structured interviews was chosen to record the diversity of experiences, inner convictions, feelings and attitudes from different perspectives while trying to answer the research questions. To make our research process more transparent a completed COREQ checklist is given in Additional file 2.

A heterogeneous group of interviewees was compiled by means of theoretical sampling [36] in order to depict the complexity of the implementation of intra- and interpersonal competences (IICs) with the most varied perspectives. We wanted to capture shared ways of thinking and deep-seated, perhaps even partly preconscious assumptions within medical education and practice. As these traits are sometimes less visible if the interviewees are part of the medical community, we also wanted to include views from people who had a different professional socialization, especially in the fields that provide more in-depth IIC training.

When selecting the physicians, an important aspect of sampling was to get a meta-level perspective of medical culture in general by including a heterogeneous view from different stakeholders and specialties. We therefore considered both operative and conservative specialities plus people from research, training and practice. To gain different perspectives, especially of IIC training, we tried to include people from medical fields that are known for their focus on IICs, such as psychiatry and psychotherapy, as well as fields that do not define themselves with this focus. Besides physicians, the study included people from other medical professions such as nursing, health advice, health politics, health insurance, and training and research in the field of communication.

To gain a non-medical perspective on medical teaching and practice, we chose non-medical professions which have a strong emphasis on IIC-training. Interviews were held with individuals from the fields of teaching, health journalism, social work, aviation, politics and business consulting. These non-medical experts also had experience within the health field. Some of these experiences were gained from being patients or supporting relatives through the system. However, most participants also had professional contact with physicians: for example, the politician was responsible for providing the patients’ view to the Federal Government, the aviation expert trained physicians in interpersonal competences, the psychotherapist had professional contact to physicians, etc. Most interviewees came from Germany, however many of the German interview partners have been working in international contexts. The study also included international perspectives with individuals from Belgium, Austria, Israel and the USA. Interviews were conducted with women and men with varying professional experience (from students up to very experienced practitioners). Details of the demographic factors are given in the results section (Table 1). In addition, the selection of interviewees was repeatedly discussed and expanded until the material represented in the qualitative data reached content saturation.

**Table 1:** Professional background of the interviewees (may include double entries)

Patients and patients’ relatives	10
Physicians	9
Medical students	2
Patient representatives (policy, advice)	2
(Medical) Teachers	3
Nursing	2
Journalism	1
Social work	1
Aviation	1
Business advice / law	1
Health insurance	1
Psychology	1

The interview guide was developed by the authors of the study based on the research questions as well as on existing literature and tested by means of two think-aloud interviews. Ambiguous questions and redundancies were clarified, and the guide revised accordingly. It was then translated into English and edited by a native translator. The definition of IICs and their relation to the “arts’ component” of medicine used in the interviews were clarified with the interviewees before the interviews started. After carrying out the interviews the researchers came across the definition of IICs used by the National Academy of Science, which matches with the definition used in the interviews in its core statements. On account of this, the definition used by the National Academy of Science has been used to discuss the main results, even though it was not used to create the interview guide. This is the reason that the examples given to illustrate IICs may differ in some points. Nevertheless, the definitions are combinable because the definition used in the interview guideline did not claim to be comprehensive but aimed to provide understandable examples of IICs for the interview partners. The complete guide can be seen in Additional file 1.

While carrying out the interviews and analysing the material the categories brought up by the interviewees suggested a division of the different research questions into two parts. While this paper will answer the first part of the interview guideline questions, a second publication will deal with the second part of questions.

The study involved a total of twenty interviews with 21 respondents (one double interview) and took place between June 2016 and March 2017, after the interviewees had been informed about the interview procedure and asked to confirm their consent to participate in the study in writing.

The ethics commission of the University of Witten/Herdecke ruled on 29 August 2016 (application number 120/2016) that there was “no ethical or legal professional objection” in relation to carrying out the study.

The interviews were carried out in a semi-structured procedure using the guideline. They were conducted in German or English, depending on the native language of the interviewee, by one of the three researchers (GL, JE, LL), face to face or on the telephone.

The interviewees were asked to answer the key questions both from their professional viewpoint and to include their perspective as patients or relatives of patients. For this reason, no separate interviews were carried out with patients.

The recorded interviews were then transcribed and anonymised. All the German quotations used in the paper were translated by a native English-speaking translator.

The transcribed interviews were read separately by all researchers and then analysed based on the *grounded theory* according to Strauss [36, 37]. This comprises the following stages: after reading, the interviews were first coded in an open manner into Max QDA, a software for qualitative text analysis, by two of the researchers (GL, LL). Following this, these two researchers developed preliminary axial coding. The third researcher, who was not involved in the open coding process and therefore not so deeply immersed in the material, had the task of looking in particular for relationships, contradictions and emerging themes. The three researchers (GL, JE, LL) refined the codes by discussing iteratively, going back and forth between the interview material and the developing axial coding system until consensus was reached [36]. “Latent categories” [36] were then defined in order to form key categories, looking for a main category through selective coding.

During the process of evaluating the material after the main theme had emerged, the researchers looked for sensitizing theories to contrast the categories and checked the inductively obtained results deductively.

As the range of material was so great, we will focus on the current state of IIC-training and, most importantly, on the barrier analysis of the underlying difficulty in implementing IIC-training effectively. In a second article we will elaborate ways to overcome these hindrances.

## Results

A total of 15.9 h of interview material (between 16 and 85 min per interview) was analysed. Ten men and eleven women were interviewed. The age of the interviewees was between 23 and 70 years (mean 49.9 years) and their professional experience between zero and 46 years (mean 23.7 years). The interviewees came from the USA [1], Belgium [2], Austria [1], Israel [1] and Germany [16]. We included physicians from different medical fields and experts from non-medical professions. We tried in particular to interview physicians from more technically focused specialties, as we assumed that they might put less emphasis on the need for IICs in the treatment process. Details of the professional demographics are given in Table 1.

Analysing the interview material generated the following categories for answering the research question:

### Equivalency of “science” and “art”

“Artistic skills”, in other words, the ability to find the best individual solution or fit for the patient from the medical knowledge by using IICs was evaluated as being at least of equal importance to the scientific component in treatment success. Some interviewees even described the artistic aspect as more important, as it allows the adjusted application of medical knowledge in each individual case. However, both components were usually found to be so closely linked that they could not be separated.

*"Even if I have the best science but cannot bring artistic skills to the man, then the science won't help me. So, it is the combination of the two."* (Aviation training manager)

*"I would not rate the purely scientific element so highly, but rather the artistic aspect of doing the right thing in relation to the patient based on the scientific knowledge."* (Specialist for psychosomatic medicine and medical teacher)

### The need for development despite diversity

The interviewees described a great individual range in the level of IICs in physicians. They described physicians who were naturally endowed with high competences. At the same time, many experiences were reported where inadequate IICs led to a lack of interpersonal fit. This lack seems to lead to disturbances within the medical team, in physician-patient relationships, in patient safety, in the practical teaching of IICs for medical students and in decreased physicians’ satisfaction. Therefore, and in comparison, to other professional groups, a general high developmental need was identified which, however, is not given enough attention during studies or in training.

*“Well, my experience is that some physicians have a high level of competences there, other physicians less. I often get the impression that training in these competences can be traced back to their personal knowledge and the value they place on these things, because dealing with these things is very rarely a course requirement.”* (Specialist for neurology)

*"[ … ] if you're lucky and come across good consultants or senior consultants who take you on their rounds, you can learn something and so on. But that's of course no guarantee that all physicians are trained to the same degree in this competence. It's then unfortunately just a matter of chance, I believe."* (6^th^ year medical student)

#### The defensive attitude against mistakes and learning

A principle result of the study is a defensive attitude amongst medical students and physicians towards their own mistakes and deficits which has been identified as the central underlying obstacle to the implementation of IICs in medical education and practice. In contrast to this attitude, in almost all the interviews the ability to deal openly with deficits and conflicts as well as to admit personal challenges or even mistakes was seen as an important element in the training of IICs. This ability is experienced as not being well enough developed in medical students and physicians. Mistakes, deficits, conflicts and interpersonal challenges tended to be hidden and perceived as something negative. The interviewees described how this enabled the emergence of an expert identity and professional self-image which was defined by the desire for control, a display of outward certainty, the subordination of your own needs and the pursuit of efficiency and perfection. For this reason, reflection on intrapersonal and interpersonal challenges and on personal emotions, motives and values was not seen as being part of the medical professional image.

The interviewees described how physicians define themselves primarily through objective knowledge and can rise in the hierarchy and further their careers by gaining this objective knowledge. In this attempt, any debate about diversity of perspective, ambivalence or uncertainty was viewed as rather a disruption and felt to be unimportant. It was stated that this attitude in medicine in turn attracts people who are in search of certainty and control. The defensive culture in medicine is thus maintained.

*“I think physicians do not usually perceive them* [IICs] *as part of their professional identity.”* (Business advisor and lawyer)

*"That's what I mean, that these are the sort of people, [...] who place a lot of importance, for themselves as well, on having things cognitively under control. You might say it's the control mechanism from which they obtain security. And this [...] then applies to the surrounding social processes as well [...] and there, mistakes are certainly something very unsettling. Of course, learning also takes place here, but it tends to be an evolutionary, unconscious process, where you always appear competent, at least to yourself and your own surroundings. People also learn, but I always say, they go down into the cellar to learn where no one is watching them."* (Business advisor and Lawyer)

*“I think, [ … ] this is related to a questionable right and wrong and that revealing your developmental processes, your vulnerability, your incompetence and your ability to deal with this incompetence [ … ] is just not part of this and does not seem to be wanted either.”* (Specialist for neurology)

#### Reasons for a defensive attitude in medical students and physicians

After mentioning the defensive attitude as a key barrier, our interviewees also mentioned different factors as to why this defensive attitude might be especially prominent in medicine.

The first aspect hindering personal reflection and development was described as the proximity of medicine to possible death or physical and mental harm. This proximity produces a high degree of responsibility linked to the physician’s profession. Our participants described a lack of support within medicine for dealing with this responsibility. This lack leads in turn to a personal emotional overload and uncertainty, accompanied by fear, shame and blame. Rather than being addressed openly, this uncertainty tends to be neglected and hidden behind an expert identity.

*"I think that dealing with shame and blame is much, much harder in medicine than in many other fields, because the responsibility is so great."* (Specialist of psychosomatic medicine and medical teacher)

*“Just think what medical students are confronted with at times. How should they process all that and deal with it when they do not have the opportunity to simply talk about it and just let it out in a professional discussion with others?”* (Nurse)

*" Physicians are afraid of mistakes."* (Specialist for neurology)

In our interviews, another factor that influences medical students and physicians as well as medical culture as a whole was described as the image of medicine as a natural science and the idea of being able to divide medical contexts into the categories of “right” and “wrong”. On the one hand, this idea of course fosters the desirable expert identity. On the other hand, the categories “right” and “wrong” are at variance with uncertainty, ambiguity and a diversity of perspectives that are highly prevalent in medical care and are needed to deal with fear, deficits and mistakes in order to grow on a personal professional level.

*“[ … ] I often notice that someone is hiding behind some scientific position or other, but that the dialogue is actually missing.”* (Physician and patients’ representative)

*"It is part of the basic socialisation [in psychology] that you learn from the start that you can look at it like this and you can look at it like that, so from the start you learn a diversity of perspectives, while I think in medical studies you tend to learn that that's how it is and that's how it should be done. From that point of view, I think that the socialisation conditions for physicians are a bit less favourable."* (Psychologist and psychotherapist)

Another factor fostering a defensive attitude towards learning from mistakes and deficits was identified in our interviews as being pressure of different kinds. This includes time, hierarchical, economic and competitive pressures. These different types of pressure prevent dealing openly with mistakes and uncertainty at different levels. On the one hand, time and economic pressures discourage the implementation of time allocated to feedback, supervision and individual meetings and training. This leads to the situation where subjects that need more space for reflection and training – e.g. IICs – are neglected and left to autodidactic efforts. On the other hand, the lack of a healthy team structure with a low hierarchy makes it hard to deal with one’s own uncertainties and mistakes in an open manner in order to allow reflection and growth on a personal level.

*"In places where strict hierarchical structures [...] are standard, handling errors is generally similarly beset by problems [...]."* (Physician and patients’ representative)

*"This is due simply to the paternalistic character of medicine. There is someone who knows a lot and he tells the other person, who has no idea, what they should do."* (Physician and patients’ representative)

*“You are constantly awarded for being fast and solving the problem as quickly as possible*.” (Physician and medical teacher)

*"Time is also a considerable factor, although time alone does not do anything, you also have to want to fill it, you have to want to talk to each other."* (Nurse and teacher in nursing school)

*"One thing is, to practise it [IICs] regularly [...] just the way I learn ECG [...] so that it's simply an important fundamental element."* (Physician and medical teacher)

## Discussion

By looking at the current state of research on the implementation of intrapersonal and interpersonal competences (IICs) in practice and training, this implementation seems to be both necessary and feasible. However, in many places there still seems to be a reluctance to implement IIC trainings, especially in the clinical part of medical school and training. Our study examined the impact of IICs (“art”) and “science” in the treatment process, the current status of IIC-teaching and, in particular, the underlying barriers to implement structured IIC development programmes in medical practice.

In order to analyse these factors from different perspectives and against the background of experiences in other disciplines that have already implemented more structured IIC training, the methodology selected was a qualitative analysis of interviews with experts from within the medical field, but also from the outside, from non-medical fields, who have had a private and/or personal insight into medical practice.

### Equivalency, diversity and the need for development

While designing the research, the assumption based on literature and researchers’ personal experiences was that “art” – i.e. IICs – and “science” are both important during the treatment process. As the first finding of this study, the interviewees confirmed this impression. The two components, art and science seldom exist in isolation but form an inseparable unit when treating patients. The inseparable connection of “art” and “science” in medicine is repeatedly described as, for example, “two sides of a coin” or “art” as “integral to medicine as an applied science” [3, 4].

As a second finding, the participants also confirmed the perception of a broad inter-individual range of these competences. Although there are positive examples of the longitudinal implementation of IICs in medical education described by our interview partners and found in the literature – e.g. the New Pathway Program at Harvard University [38] – a general need for development has been identified. Overall it appears that the training of IICs demanded in our study and in the literature is not yet adequately implemented in the sense of a thorough personal learning during medical education, so that in many cases it does not lead to sustainable competences [9, 10]. Studies have shown that these competences tend to be conveyed by chance and autodidactically [13]. This is the case despite growing evidence within medicine and other professional fields indicating that the longitudinal implementation of IIC training is possible [1, 38] and that adequately developed IICs lead to a decrease in patient risk, an increase in physician satisfaction and better outcomes [17, 21].

To understand why reflection on and teaching of IICs is not yet implemented in a structured way in medicine, this study also looked at the state of implementation of IICs in other professions. In other professional fields such as e.g. nursing or teaching, there are examples of a structured implementation of IICs in education and training [39, 40]. In medical education the teaching of personal professional competences is to date provided mostly through the teaching of single competences in preclinical courses such as e.g. communication outside the clinical context [41, 42]. The main target is the provision of skills and feedback [43]. In practice these competences (communication, feedback, professionalism, resilience, empathy etc.) occur conjointly and are developed into expertise through reflection and feedback on complex practice situations. The human factor in medical errors has just started to be acknowledged to be a target in medicine on the individual and the systemic level [44]. In aviation, this factor is addressed as an important element for greater safety: a safety training called *crew resource management* was established and flight accidents were reduced significantly [28, 29]. Recently this training was adapted and used to train physicians in orthopaedics and traumatic surgery in interpersonal competences as well as in mindsets so that errors can be reflected and discussed honestly and openly within the team [45]. Despite these positive approaches to a systematically implementation of medical IIC-training there longitudinal training programmes are still not the rule, especially in clinical settings [10, 38].

Our main research question asks why there seems to be a reluctance to implement the training of IICs in the clinical part of education and training in many places, if they are known to be important in the treatment process and there are examples of improvement after their implementation.

### A defensive attitude as a key obstacle for the implementation of IICs

While following up our research questions, the interviewees pointed towards shared ways of thinking and deeper shared assumptions which they felt are hindering factors in the implementation of IIC training. These factors are normally hard to capture and can be subsumed as a defensive attitude towards the open handling of personal deficits, uncertainty and mistakes. As the implementation of IICs needs to involve reflection and feedback on personal characteristics such as emotions, motives and values, this defensive attitude is identified as the key factor hindering this implementation and is therefore the new and main finding of this study.

During the evaluation of the interview material this defensive attitude towards learning from mistakes and deficits, especially on an intra- and interpersonal level, reflected the *fixed mindset* described in Carol Dweck’s mindset theory [32]. This concept proved to be a suitable sensitising concept for the repeated contrasting and elaboration of the inductively obtained results deductively.

The *fixed mindset* describes the attitude of a person who sees competence as something static, a given which leads to the desire to appear outwardly competent [32]. As a result of this attitude people avoid challenges, give up quickly, perceive their efforts as unproductive, ignore constructive critical feedback and are intimidated by others’ successes, so that their further development is limited [32]. Dweck was able to demonstrate that a *fixed mindset* is an important barrier to learning processes in children. The commonality between our finding and the *fixed mindset* concept is that this adverse attitude towards being open to learning is perceived as a major obstacle to progress. The expert identity described by our participants has similar characteristics to Dweck’s *fixed mindset*. One difference between our findings and Dweck’s concept of the *fixed mindset* is that our defensive attitude was also described in the context of personal learning. Here there seems to be a notion that there is no need to question oneself and to further develop ones IICs. A second difference is that, in medical training and practice, this defensive attitude does not seem to be solely an individual defensive approach to challenges which limits the further development of individual players, but also a basic cultural factor which has a personal but also curricular and institutional effect on training and practice in medicine.

The question remains as to why medical education and practice are particularly receptive to this *fixed mindset* culture in comparison to other professions. It is important to discover the reasons for this in order to design effective implementation strategies.

### Reasons for a fixed mindset culture

The interviewees describe the current culture in medicine as a *fixed mindset* culture due to several factors which hinder reflection, feedback and development on a personal level. They describe physicians overloaded with a high responsibility through being at risk of causing physical or mental harm related to their work. They also perceive the lack of a constructive handling of negative feelings such as uncertainty, fear and shame. They describe the lack of the inclusion of uncertainty, ambivalence and perspective diversity in a culture of right and wrong decisions, based on the assumption that medicine is mainly a natural science. A third factor leading to a fixed mindset culture was identified as a lack of time, space and a healthy and supportive team structure that allows reflection, feedback and supervision.

The negative environmental factors fostering the *fixed mindset* culture such as hierarchy, poor communication and disruptions to cooperation in the team are also described in other studies [46]. For instance, the difficulties in dealing with mistakes, the aversion to including IICs as part of error communication, and an unconstructive hierarchy have already been described as a negative influence on teamwork. Reciprocal feedback was identified as a positive factor on a healthy working atmosphere in e.g. operating theatres and intensive care units [21, 42]. Furthermore, irregularities in communication and cooperation in unsupportive team structures were identified as a source of disruption to patient safety and to patients’ and physicians’ satisfaction [19, 23, 43].

As we also wanted to include the experiences of other professional fields, we looked at how these barrier factors are dealt with in the education of other professions. As our psychological participant stated, psychology includes a diversity of perspectives and therefore the tolerance of uncertainty in a structured way: “It seems neither desirable to restrict diversity [...] to a ‘single model’ nor conceivable that there could or should be only a single view of psychological defects [...]” [44]. In nursing, teaching and management professions emotional and social competences are also recognised as essential for professionality while experience and reflection are named as being important to train these competences [24–27, 30, 31]. Further, safety strategies in aviation are a fruitful example of the modification of hindering factors. In aviation the influence of hierarchy was examined, and an open feedback culture developed and implemented. By training rigorous feedback structures in daily practice in a reciprocal manner they were able to significantly decrease the occurrence of “critical safety situations” [29].

By identifying the hindering factors named by our interviewees and with positive examples from medical and non-medical fields in mind, the above-mentioned shortcomings can form a starting point for creating a personal mindset and institutional culture for implementing IICs. In order to change attitudes and cultures in favour of longitudinally structured IIC education and training in more institutions, the underlying barriers as well as the mindset of defensiveness to learning from mistakes and deficits must be addressed. This could allow a change that goes beyond the provision of single skills.

Addressing these deeply rooted barriers and asking ourselves as individuals and organisations whether our mind is fixed in order to strengthen the “arts part” of medical treatment seems an important lever to improve the quality of care for individual patients, teams and organisations.

However, the first step must be to find a solution for changing these underlying barrier factors. A second paper will deal with the second part of the interview material. It will focus on the steps necessary to address these barriers in order to produce more sustainable change.

## Conclusion

The study showed that, by neglecting the *person* and their IICs in medical training, the important element of medical artistic skills is not adequately developed, which can result in a decrease in quality at different levels. Key barriers in this are the defensive attitude to addressing challenges, learning and mistakes regarding IICs which characterises medical training and practice. The combination of personality-related and environmental factors maintains this attitude. This attitude does not just apply to individuals in medicine but tends to define the culture of the professional field. From these interrelationships it became apparent that change needs to be addressed on a personal level but probably even more so on a cultural one.

### Advantages and disadvantages of the study

In addition to various studies which address the teaching of some aspects and single competences, this study is, to our knowledge, the first that explicitly focuses on the question as to why, compared to other vocational fields, IICs in general are not yet implemented adequately in terms of structured longitudinal set-ups. Since in practice the different competences summarised under the umbrella of IICs have to be handled conjointly, this broad general view seemed adequate for looking for a common underlying obstacle to the implementation. In addition, through the selection of its interviewees, it was the first study to include people from non-medical professions but possessing experience with medicine and with multinational backgrounds referring to the multidisciplinary status of IICs. This approach was chosen to provide a perspective on medical culture from outside the medical field because other professional fields already implement IICs more systematically and in a longitudinal manner. By being receptive to perspectives from other vocational fields and working on broad research questions in a reflective setting, this study was able to capture a meta-level perspective on the research topic. This perspective enabled the interview partners to mention these underlying assumptions and attitudes that emerged as hindering factors, providing the key finding of this study.

One drawback of the study, however, is that it dealt exclusively with people from western states and the experiences from other cultural contexts are therefore overlooked. In addition, although all the interviewees were urged to include their patient perspective in their answers, no one was asked solely about the patient perspective.

While designing and carrying out our research we constantly reflected on the researchers’ assumptions and personal, professional and theoretical backgrounds. To be able to identify our own bias, one of the researchers was not included in the coding process so that they were able to provide a critical view of the findings.

Although when selecting the interviewees, we attempted to obtain a heterogeneous sample and to include experts from technical medical fields as we had anticipated an attitude which would lead them to view the teaching of IICs as of minor importance, this was not reflected in the interview material. This could be since our sampling was not heterogeneous enough. But it could also be viewed as an indication that the lack of these competences is perceived in general but not as easily in oneself.

### Summary and outlook

This study was designed to describe the relevance and the status quo of the implementation of IICs in medical education and practice, and to name reasons for the assumed comparatively poor implementation of IICs. The main element that emerged from pursuing these research questions was a defensive attitude towards intra- and interpersonal learning in medical culture that was described as a key element preventing the development of IICs. Furthermore, the interviewees found shared ways of thinking and deeper shared assumptions which promoted this defensive attitude: first, a lack of support in handling negative feelings such as uncertainty, fear and shame in the context of a high level of responsibility, and the risk of causing physical or mental harm; second, the exclusion of uncertainty, ambivalence and perspective diversity in a culture of right and wrong decisions; and third, environmental factors such as a lack of time, space and a healthy and supportive team structure that allows reflection, feedback and supervision. By identifying these factors, they can be addressed to improve the implementation of IICs.

The second part of our work will focus on practical interventions that can change this defensive attitude at different levels of medical teaching and practice.

## Additional files

Additional file 1: Questionnaire: Intra- and interpersonal competences in medicine. (PDF 102 kb)

Additional file 2: Consolidated criteria for reporting qualitative studies (COREQ) checklist (PDF 190 kb)

## Data Availability

The datasets used and analyzed during this study are fully available from the corresponding author at any time. Though, the authors decided not to include the whole data within this paper due to the fact that most data material collected in German language.

## Abbreviations

IICs: intra- and interpersonal competences
