# Supplementary material for: Is your mind set? – how are intra- and interpersonal competences dealt with in medical education? A multi-professional qualitative study
Source: BMC Med Educ. 2019 Aug 22;19:317. doi: 10.1186/s12909-019-1748-y (PMC6704522; doi:10.1186/s12909-019-1748-y)
Supplement: Supplementary file 1 — Questionnaire: Intra- and interpersonal competences in medicine. (PDF 102 kb) [file 12909_2019_1748_MOESM1_ESM.pdf]

Questionnaire: Intra- and interpersonal Competences in Medicine

Demographic factors:

Task/Job description

Age

Gender

Professional years

Theoretical background

Educational background

Interview Guide

We would like to ask you questions about the development of intra- and interpersonal skills and attitudes in medical education and in Your field of expertise.

Are the terms intra- and interpersonal competences (skills and attitudes) clear?

1. **How we define intrapersonal competences (skills and attitudes):**
   Intrapersonal competences reflect skills and attitudes towards oneself, including self-awareness, the ability to reflect, self-regulation, self-care, moral resilience, and the ability to develop ideals and values that stimulate the development of professional attitudes as well as the pursuit [of] lifelong learning.
2. **How we define intrapersonal competences (skills and attitudes):**
   Interpersonal competences are skills and attitudes that promote responsible and educational relationships and include patient-centeredness, empathy, and constructive relationships with patients, colleagues and institutions and the management of errors.

We will ask you about the education of intra- and interpersonal competences

a. **In the area of your professional expertise**
b. **In relationship to how patients perceive physicians intra- and interpersonal competences**

We will ask you to evaluate the educational situation regarding intra- and interpersonal competences with Your background of expertise:

3. Intra- and interpersonal skills and attitudes are needed to provide the most effective treatment to individual patients in a given, clinical situation. These competences are likely addressed when one talks about medicine as a science and an art. How do science and art come together to enable the provision of qualitative, patient care? (What would medicine be without one or the other?)

4. What do you think about the current state of intra- and interpersonal skills and attitudes among physicians? In relationship[s] to:
    a. patient communication?
    b. interprofessional communication?
    c. quality management?
    d. self-awareness, self-regulation, self-care?

5. What would be different if intra- and interpersonal expertise among physicians were better? What would be different:
    a. for patients?
    b. for co-workers and teamwork
    c. regarding individual job satisfaction?
    d. for health-care institutions?

6. **What is, in your opinion, the economic impact of poor intra- and interpersonal skills and attitudes?**

7. **Under the given economic circumstances of the health care system (high workload, high administrative burden, time constraints, and economic pressure), how do You think intra- and interpersonal competences can be developed and sustained?**

8. **How can intra- and interpersonal competences be effectively developed and integrated into the medical school curriculum?**
    a. What is necessary to develop intra- and interpersonal skills and attitudes?
    b. How is it possible to assess intra- and interpersonal competences given “assessment drives learning”?

9. **There is a debate about whether intra- and interpersonal competences are state or trait. What do you think about this debate? Can the aforementioned competences be developed in medical school or are they relatively stable?**

10. **In every discipline, expertise (like driving a car, playing the piano, perform surgery, etc.) are gained through knowledge provision, skill-building and supervised experience. Why do you think the supervision and feedback of intra- and interpersonal competences at the workplace is not prioritized in medical education? What do you think can be learned through skills training with simulated patients; and What can be learned through reflection and feedback at the workplace?**

11. **What are the pros and cons of including or excluding professional reflection or supervision in/from medical education?**

Thank you very much for agreeing to participate in this interview!
