# Supplementary material for: Is your mind set? – how are intra- and interpersonal competences dealt with in medical education? A multi-professional qualitative study
Source: BMC Med Educ. 2019 Aug 22;19:317. doi: 10.1186/s12909-019-1748-y (PMC6704522; doi:10.1186/s12909-019-1748-y)
Supplement: Supplementary file 2 — Consolidated criteria for reporting qualitative studies (COREQ) checklist (PDF 190 kb) [file 12909_2019_1748_MOESM2_ESM.pdf]

Consolidated criteria for reporting qualitative studies (COREQ) checklist

- **Domain 1: Research team and reflexivity**
  - **Personal characteristics**

1. **Interviewer / facilitator: Which author/s conducted the interview or focus group?**
   The interviews were conducted by Jan Ehlers (two interviews), Gabriele Lutz (15 interviews) and Lisa Lombardo (three interviews).
2. **Credentials: What were the researcher's credentials?** E.g. PhD, MD
   Jan Ehlers has a DVM and PhD, is a professor, leads an institute for medical education research and is vice-president of the university. Gabriele Lutz has an MD and PhD. She is a physician for neurology and psychosomatic medicine, teaches reflection and personal professional development and is a researcher in that field. Lisa Lombardo is a PhD student and is involved in organising a mentoring programme.
3. **Occupation: What was their occupation at the time of the study?**
   Jan Ehlers was vice president of Witten/Herdecke University and chair for Didactic and Education Research in Health Care at the Faculty of Medicine of Witten/Herdecke University. Gabriele Lutz was a physician working at the department of psychosomatic medicine at the Gemeinschaftskrankenhaus Herdecke and had a research position at the Integrated Curriculum for Anthroposophic Medicine (ICURAM) at the chair for Medical Theory, Integrative and Anthroposophic Medicine at Witten/Herdecke University. Lisa Lombardo was a clinical year medical student and PhD student.
4. **Gender: Was the researcher male or female?**
   Two of the researchers were female and one was male.

5. **Experience and training**: What experience or training did the researcher have?

   Jan Ehlers has a training as a veterinary surgeon, a medical educationalist and in technology enhanced learning (TEL). He has an additional specialisation in informatics. He has long-standing experience of over 20 years in teaching medical education and in higher education didactics as in TEL. Gabriele Lutz has a training as a neurologist and as a specialist in psychosomatic medicine. She also has long-standing experience in teaching reflection and personal professional development and has conducted research in this field for about 10 years. Lisa Lombardo had finished three years of medical education at the time the research project started. She was and still is organising the mentoring programme at Witten/Herdecke University, providing a reflection setting for all medical students. Besides medical school, she holds reflection workshops for German volunteers abroad.

- **Relationship with participants**

1. **Relationship established: Was a relationship established prior to study commencement?**

   Some of the participants were known by the researchers from clinics, medical school or congresses before carrying out the interviews, others were unknown and chosen because of their backgrounds. There were no work affiliations related to the research questions with any of them and therefore no bias in terms of common interests. Participants were chosen to obtain heterogeneous perspectives on the research questions.

2. **Participants knowledge of the interviewer: What did the participants know about the researcher?** E.g. personal goals, reasons for doing the research

   The participants were informed of the reasons for doing the research. None of the interviewers hid their opinions or goals but encouraged

participants to speak out and were eager to hear about their feelings, perceptions and judgement.

3. **Interviewer characteristics: What characteristics were reported about the interviewer / facilitator?** E.g. bias, assumptions, reasons and interests in the research topic

   Jan Ehlers did not have much experience with teaching IICs in medical education but had a rather neutral stance. He therefore had a more reflective, monitoring position in the research process in order to reflect biases and confounding assumptions.

   Gabriele Lutz has a strong focus on improving IICs with her teaching and carries out research to promote this field. There was therefore a bias which has been reflected throughout the research process.

   Lisa Lombardo is a research student who is critical about some aspects of IIC teaching in Germany and is co-organising a mentoring programme to improve personal professional development in medical students. She therefore also has a bias but was very open towards the research findings and aware of her assumptions. She also was aware of her beliefs throughout the research process.

- **Domain 2: study design**
  - **Theoretical framework**

1. **Methodological orientation and theory: What methodological orientation was stated to underpin the study?** E.g. grounded theory, discourse analysis, ethnography, phenomenology, content analysis

   Grounded theory was used to analyse the data.

  - **Participant selection**

1. **Sampling: How were participants selected?** E.g. purposive, convenience, consecutive, snowball

   The participants were selected purposively and consecutively until theoretical saturation had been reached.

2. **Method of approach: How were participants approached?** E.g. face-to-face, telephone, mail, email

   Most participants were approached by e-mail. A few were approached face-to-face.

3. **Sample size: How many participants were in the study?**

   There were 21 participants in 20 interviews (one double interview) in the study.

4. **Non-participation: How many people refused to participate or dropped out? Reason?**

   There was no non-participation or drop-out.

- **Setting**

1. **Setting of data collection: Where was the data collected?** E.g. home, clinic, workplace

   The data were collected in different places. Interviews were conducted personally or over the phone, depending on the distance between interviewer and interviewee. Interviews took place in the workplace, at the interviewee’s home and at the medical school and one was conducted at a conference location.

2. **Presence of non-participants: Was anyone else present besides the participants and researchers?**

   There was no one else present besides the participant and the researcher.

3. **Description of sample: What are the important characteristics of the sample?** E.g. demographic data, date

   The interviews were carried out between July 2016 and March 2017. Ten men and eleven women were interviewed. The age of the interviewees was between 23 and 70 years (mean 49.9 years) and their professional experience between zero and 46 years (mean 23.7 years). The interviewees came from the USA (1), Belgium (2), Austria (1), Israel (1)

and Germany (16). There were 9 physicians, 2 medical students, 2 patient representatives, 2 teachers, 2 nurses and one participant from each of the fields of journalism, social work, aviation, business advice / law, health insurance and psychology. Other characteristics are described in the results section.

- **Data collection**

1. **Interview guideline: Were questions, prompts, guides provided by the authors? Was it pilot tested?**

   In the interview guide questions, prompts and guides were provided. We performed two think-aloud interviews in order to test and adjust the guides appropriately.

2. **Repeat interviews: Were repeat interviews carried out? If yes, how many?**

   No, there were no repeat interviews.

3. **Audio / visual recording: Did the research use audio or visual recording to collect the data?**

   The data was collected on audio.

4. **Field notes: Were field notes made during and / or after the interview or focus group?**

   No, no field notes were made.

5. **Duration: What was the duration of the interviews or focus group?**

   In total 15.9 hours of interview material was collected (between 16 and 85 minutes per interview).

6. **Data saturation: Was data saturation discussed?**

   Yes, interviews were carried out until all three researchers felt that data saturation was reached. As described in the results section when describing the research process, we had the feeling that we had an emphasis on the importance of IICs in the treatment process and on the

need to develop them. We therefore included two more interviews with medical experts from more technically oriented specialisms. In contrast to our assumption, they confirmed the existing categories and we therefore concluded that we had reached data saturation.

7. **Transcripts returned: Were transcripts returned to participants for comment and / or correction?**

   No, the transcripts were not returned to the participants.

- **Domain 3: Analysis and findings**
  - **Data analysis**

1. **Number of data coders: How many data coders coded the data?**

   There were two data coders.

2. **Description of the coding tree: Did the authors provide a description of the coding tree?**

   No, but we can provide it on request.

3. **Derivation of themes: Were themes identified in advance or derived from the data?**

   The themes were derived from the data.

4. **Software: What software, if applicable, was used to manage the data?**

   MaxQDA was used to manage the data.

5. **Participant checking: Did participants provide feedback on the findings?**

   No, the participants did not provide feedback on the findings.

  - **Reporting**

1. **Quotations presented: Were participant quotations presented to illustrate the themes / findings? Was each quotation identified?** E.g. participant number

Yes, quotations were presented which have been anonymised by the researchers in such a way that they can be re-identified. Within the text we provided a brief identifier for the reader, so she/he can relate the quote to the expertise of the interviewee.

2. **Data and findings consistent: Was there consistency between the data presented and the findings?**

   Yes, there was.

3. **Clarity of major themes: Were major themes clearly presented in the findings?**

   We hope so. In response to the reviewers' comments, we have added some more quotes to substantiate the findings.

4. **Clarity of minor themes: Is there a description of diverse cases or discussion of minor themes?**

   Yes, for example interviewees said that there are many physicians who have very good IICs, but that there is a general developmental need. So all in all there is a great variety.
